# Transcriptomic Analysis Comparing Tumor-Associated Neutrophils with Granulocytic Myeloid-Derived Suppressor Cells and Normal Neutrophils

**DOI:** 10.1371/journal.pone.0031524

**Published:** 2012-02-14

**Authors:** Zvi G. Fridlender, Jing Sun, Inbal Mishalian, Sunil Singhal, Guanjun Cheng, Veena Kapoor, Wenhwai Horng, Gil Fridlender, Rachel Bayuh, G. Scott Worthen, Steven M. Albelda

**Affiliations:** 1 Institute of Pulmonary Medicine, Hadassah-Hebrew University Medical Center, Jerusalem, Israel; 2 Thoracic Oncology Research Laboratory, University of Pennsylvania, Philadelphia, Pennsylvania, United States of America; 3 The Wistar Institute, Philadelphia, Pennsylvania, United States of America; 4 Division of Neonatology, University of Pennsylvania, Children's Hospital of Philadelphia, Philadelphia, Pennsylvania, United States of America; Istituto Superiore di Sanità, Italy

## Abstract

The role of myeloid cells in supporting cancer growth is well established. Most work has focused on myeloid-derived suppressor cells (MDSC) that accumulate in tumor-bearing animals, but tumor-associated neutrophils (TAN) are also known to be capable of augmenting tumor growth. However, little is known about their evolution, phenotype, and relationship to naïve neutrophils (NN) and to the granulocytic fraction of MDSC (G-MDSC).

In the current study, a transcriptomics approach was used in mice to compare these cell types. Our data show that the three populations of neutrophils are significantly different in their mRNA profiles with NN and G-MDSC being more closely related to each other than to TAN. Structural genes and genes related to cell-cytotoxicity (i.e. respiratory burst) were significantly down-regulated in TAN. In contrast, many immune-related genes and pathways, including genes related to the antigen presenting complex (e.g. all six MHC-II complex genes), and cytokines (e.g. TNF-α, IL-1-α/β), were up-regulated in G-MDSC, and further up-regulated in TAN. Thirteen of the 25 chemokines tested were markedly up-regulated in TAN compared to NN, including striking up-regulation of chemoattractants for T/B-cells, neutrophils and macrophages.

This study characterizes different populations of neutrophils related to cancer, pointing out the major differences between TAN and the other neutrophil populations.

## Introduction

Various types of myeloid cells have been shown to promote tumor progression by direct immune suppression [1] and by production of angiogenic factors, matrix-degrading enzymes, or growth factors [2], [3]. The best characterized of these cells have been tumor-associated macrophages (TAM) that have properties of alternatively activated macrophages, also known as M2 macrophages [1]. We have recently shown that, similar to TAM, tumor-associated neutrophils (TAN) also have differential states of activation/differentiation. We demonstrated that in tumors, TAN develop a pro-tumorigenic (N2) phenotype, largely driven by the presence of TGF-β. Blocking TGF-β changes the characteristics of these cells to a more anti-tumorigenic (N1) phenotype [4]. IFN-β has been suggested to polarize this dichotomy in a reciprocal way, favoring the N1 phenotype [5]. In untreated tumors, neutrophils have been reported to produce angiogenic factors and matrix-degrading enzymes [6], [7], support the acquisition of a metastatic phenotype [8], and suppress the anti-tumor immune response [9]. Depletion of these TAN inhibits tumor growth [4], [6], [10] and reduces the level of immunosuppression in the tumor microenvironment, allowing for increased activity of CD8^+^ cytotoxic T-lymphocytes (CTL) [4]. The broad spectrum of activities of TAN in the context of tumor biology has been recently reviewed [11].

Neutrophils, like all other leukocytes, move into tissues from the blood under the influence of specific chemokines (e.g. KC/CXCL-1, MIP-2α/CXCL-2 and GCP-2/CXCL-6), cytokines (e.g. TNF-α and IFN-χ), and cell adhesion molecules located on their own surface (e.g. CD11b) and on the surface of endothelial cells (e.g. selectins, ICAM-1 and PECAM-1) [12]. When they traffic into tumors, they are referred to as TAN. In mice, TAN can be defined by the specific surface markers CD11b and Ly6G with low expression of macrophage markers such as F4/80 [13].

Myeloid-derived suppressor cells (MDSC) are a heterogeneous population of immune suppressive cells that are produced excessively in cancer. They comprise at least two subsets - granulocytic (Ly6G^+^) and monocytic cells (Ly6C^+^), potentially with different immunosuppressive properties [14]. It has been previously shown that MDSC can enter tumors and differentiate to mature macrophages (TAM) or neutrophils (TAN) [15]. However, since no definitive markers have been established, it is unknown whether intratumoral N2 neutrophils (N2 TAN) are granulocytic MDSC from spleen that are attracted to the tumor or if they are typical blood-derived neutrophils that are then converted to an N2 phenotype by the tumor microenvironment, specifically by the high local concentrations of TGF-β.

The purpose of this study was to use a transcriptomics approach to gain further information about TANs by comparing the RNA profile of these cells to naïve bone-marrow neutrophils (NN) and to the granulocytic fraction of myeloid derived suppressor cells (G-MDSC). We examined which pathways and gene-groups varied amongst these 3 populations of neutrophils and performed a detailed analysis on pathways related to the main functions of neutrophils, such as respiratory burst, granule proteins, phagocytosis, apoptosis, structural genes, antigen presentation and specific immune effects. Our data defines TAN as a unique population of neutrophils, quite distinct from both NN and G-MDSC.

## Results

### Preparation of samples

mRNA preparations were extracted from individual non-tumor-bearing mice (NN) or from mice in which AB12 mesothelioma tumors were growing (G-MDSC and TAN). For each experimental “sample”, equivalent amounts of mRNA were pooled from 2–4 extractions (according to the yield of neutrophils) for the transciptome analysis. We prepared multiple independent samples from each of the neutrophil populations studied - NN (n = 7), G-MDSC (n = 4) and TAN (n = 4).

### Hierarchical clustering of neutrophils

**Figure 1:**
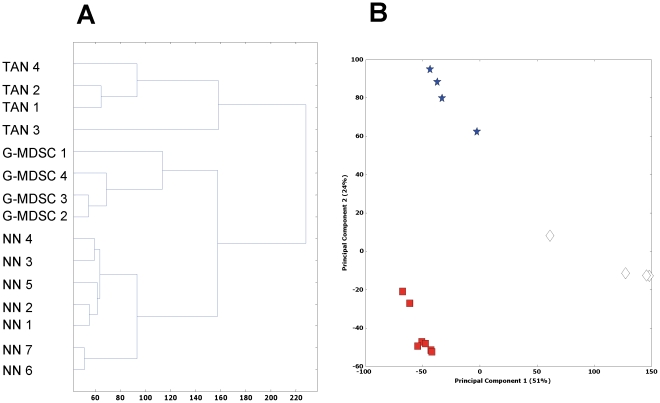
Hierarchical clustering (A) and principal component analysis (PCA) (B) of the samples, showing that the three groups of neutrophils are distinct. □ - Naïve neutrophils (NN); * - Granulocytic fraction of MDSC (G-MDSC). ◊ - Tumor associated neutrophils (TAN).

As a first step, gene probes were filtered, resulting in 12,129 informative probes. Hierarchical clustering (Fig. 1A) and principal component analysis (PCA) (Fig. 1B) showed that the three types of myeloid cells are distinct groups, with the bone marrow neutrophils (NN), granulocytic fraction of myeloid-derived suppressor cells (G-MDSC), and tumor-associated neutrophils (TAN) clearly separated. Hierarchical clustering suggested that NN and G-MDSC seemed more closely related to each other than to TAN (Fig. 1A).

### Genomewide RNA expression profiles

Whole murine genome RNA expression profiles of neutrophils from the three different groups were compared to each other to discern more specific differences. There were 8,355 genes that were significantly different when comparing NN to G-MDSC (p<0.05, FDR = 3%). The fold changes were larger than 1.7 in 2,489 of the genes. Of these genes, 1,189 were up-regulated and 1,300 down-regulated in G-MDSC compared to NN. Three of the top-5 up-regulated genes in G-MDSC were chemokines (CXCL-2, CCL-3 and CCL-4), that were changed by more than 40-fold (Table S1).

There were 11,035 genes that were significantly different between NN and TAN (p<0.05, FDR = 1%). Of these, 5,846 had a fold-change of more than 1.7-fold; 2,983 were up-regulated and 2,863 down-regulated in TAN compared to NN. Seven of the top-10 up-regulated in TAN were chemokines, all by more than 70-fold (Table S2).

There were 10,172 genes significantly different (p<0.05, FDR = 2%) between G-MDSC and TAN. Of these, 5,344 had a fold-change of more than 1.7-fold; 2,803 genes were up-regulated and 2,541 down-regulated in TAN compared to G-MDSC. Again, three of the top-5 up-regulated in TAN were chemokines (CCL-7, CCL-8 and CCL-12), all by more than 80-fold (Table S3).

**Figure 2:**
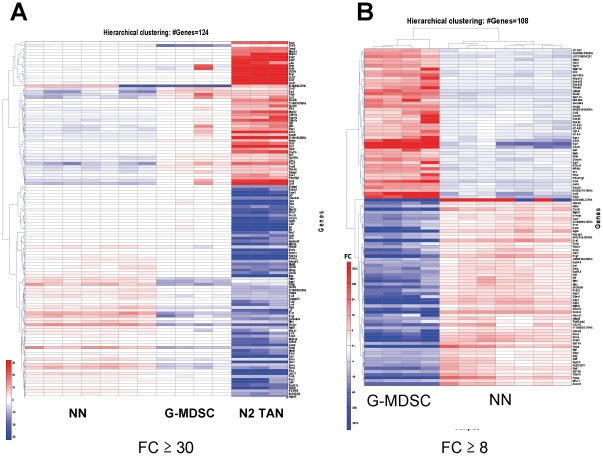
Heatmaps comparing the most different genes between the 3 groups of neutrophils. (A) – Heatmap of genes with fold-change between any two groups ≥30. The TAN exhibit a signature that is markedly different than the other two types of neutrophilic cells. (B) Heatmap with all genes with a fold-change ≥8 between NN and G-MDSC, showing a clearly different signature in these two groups of cells. Red – up-regulation; Blue – down-regulation; White – no change from mean.

In order to further compare these differences, heatmaps of the genes most changed between the groups were prepared. Figure 2A shows a heatmap including all genes with a fold-change between samples ≥30. The TAN exhibit a signature that is markedly different than the other two types of neutrophilic cells, suggesting that the TAN are not G-MDSC that have simply entered the tumor. Figure 2B compares NN and G-MDSC, showing all genes with a fold-change ≥8. It can be noted clearly that the signature of these two groups of cells differ, suggesting that the G-MDSC have changed from the bone marrow neutrophils (NN).

### Validation of gene array results by RT-PCR and ELISA

Recent technological improvements have increased the reproducibility and reliability of microarray results [16]. Most microarray results are now highly accurate, especially for highly regulated genes, and differences between microarray- and PCR-generated data occur mostly in the amplitude of the detected expression change [17], [18]. Nevertheless, we conducted real-time PCR validation of some of the gene expression changes in TAN versus NN mentioned in the results section. In addition, we validated some of the genes using cells from animals bearing a different cell line, the lung cancer LKR line, in a different mouse strain (Table 1). In general, the results were highly concordant between the RT-PCR in both cell lines and microarray data, especially regarding the direction of change in expression, although some differences in the extent of change were found, possibly related to technical differences between the methods (e.g. in MMP-9). One exception was VEGF. Whereas in the array the level of mRNA for VEGF in TAN was higher than NN, the opposite was found in RT-PCR (Table 1). We also validated by RT-PCR some of the changes in TAN versus G-MDSC. In order to show that changes are mainly due to the location of neutrophils (tumor versus spleen) and not merely the time of isolation, the confirmation of these key transcripts was done in neutrophils isolated from tumors and spleens at the same time, both at an early time point (Day 14) and at a late time point (Day 21) (Table 2).

**Table 1:** Validation of array results by real time PCR - Ratio of expression in TAN compared to NN.

*Gene (mRNA)*	*Array*	*RT-PCR*	
		*(AB12)*	*(LKR)*
ICAM-1	5.1	7.6	2.7
KC/CXCL-1	131.5	75.9	+++
MIP-2α/CXCL-2	188.7	++	+++
IP-10/CXCL-10	2.7	1.8	++
CCL-3/MIP-1α	77.6	7.7	+++
CCL-17	25.9	+++	+++
CCL-2/MCP-1	6.6	+++	ND
CCL-5/Rantes	12.3	9.6	ND
VEGF	2.9	0.5	ND
MMP9	0.37	<0.01	ND

Confirmation of selected results from the microchip array using real time RT-PCR in isolated tumor associated neutrophils (TAN) from flank tumors of two separate tumor cell lines – the mesothelioma cell line AB12 (Balb/C), and the NSCLC cell line LKR (B6-129/J1). Table shows the gene fold-change of TAN to Naïve neutrophils (NN) using the arrays or by RT-PCR measurements.

++ - Fold-change >100, +++ - Fold-change >500, ND – Not Done.

**Table 2:** Validation of array results by real time PCR - Ratio of expression in TAN compared to G-MDSC.

*Gene (mRNA)*	*Array*	*RT-PCR*	
		*(14 days)*	*(21 days)*
CCL-17	23.5	++	79.2
TREM-1	0.43	<0.01	<0.01
CD9	0.24	0.21	0.42
IL-6	2.5	8.3	1.6
VEGF	2.5	1.1	1.3
CCL3	1.3	1.4	2
CCL5	1.4	1.2	0.65
CXCL10	0.7	1.2	ND

Confirmation of selected results from the microchip array using real time RT-PCR in isolated tumor associated neutrophils (TAN) from flank tumors of two separate tumor cell lines – the mesothelioma cell line AB12 (Balb/C), and the NSCLC cell line LKR (B6-129/J1). Table shows the gene fold-change of TAN to Naïve neutrophils (NN) using the arrays or by RT-PCR measurements.

++ - Fold-change >100, ND – Not Done.

**Figure 3:**
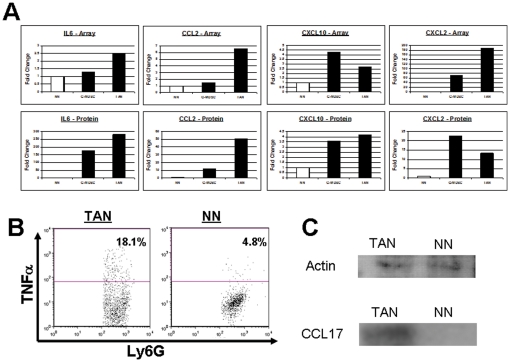
Validation of array results at the protein level. (A) – comparison between the secretions of the different cytokines to the supernatant following isolation of each of the neutrophil populations. (B) – An example of TNF-α levels in Ly6G^+^ neutrophils, comparing TAN (left)to NN (right). A clear up-regulation, similar to the increased m-RNA in the array and in RT-PCR is shown. (C) – An immunoblot, showing the expression of CCL-17 in a protein extract of TAN (left), and the lack of expression in protein extract of NN (right).

We further evaluated the protein expression levels of several cytokines and chemokines that were up-regulated in TAN, using several methods to detect proteins. We found by ELISA of conditioned media that several proteins had similar fold-changes in their levels compared to the array in the different populations of neutrophils (Fig. 3A). We also found up-regulation of TNF-α in TAN compared to NN shown by intracellular flow cytometry. On average 15.8±2.1% of TAN expressed TNF-α (n = 5), versus 4.1±0.7% of NN (n = 3). A sample dot plot is shown in Fig. 3B. Up-regulation of CCL-17 was shown by immunoblotting of isolated cells (Fig. 3C).

### Evaluation of pathway and gene groups differences

**Figure 4:**
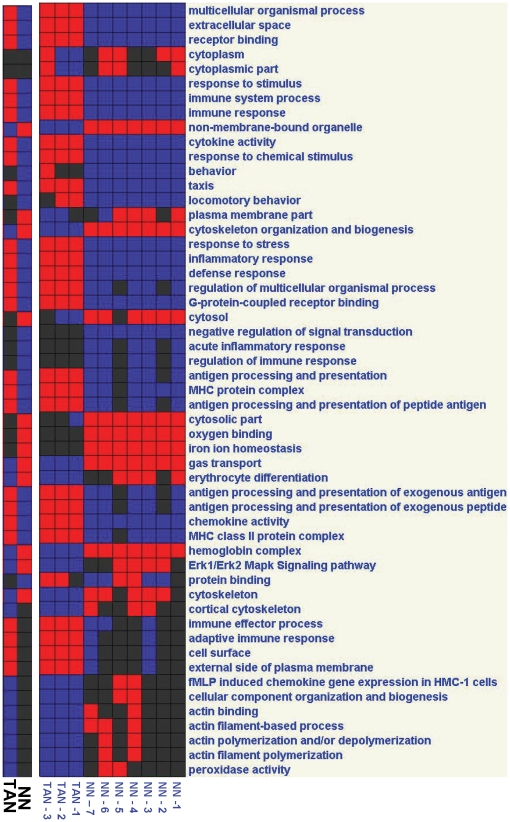
Analysis of pathways and gene groups using Genomica, comparing tumor associated neutrophils (TAN) to naïve neutrophils (NN). Each sample was evaluated for changes in the different pathways, and was marked as positive when ≥3 genes were significantly changed to the same direction. In each panel - Right - Pathways/Groups that had more than 5 samples changed. Left - Pathways/Groups that were significantly changed when the groups were compared to each other (p<0.05, corrected). Red – up-regulation; Blue – down-regulation; Black – no change from mean.

**Figure 5:**
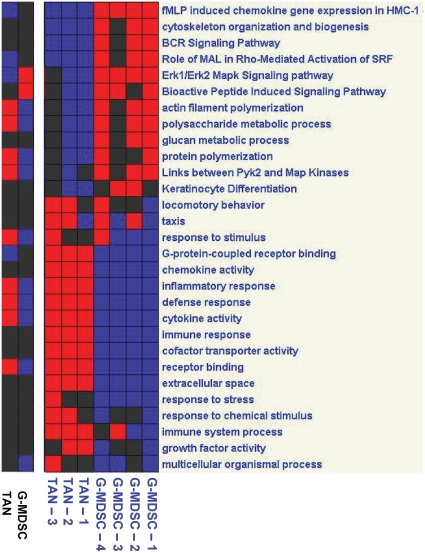
Analysis of pathways and gene groups using Genomica, comparing tumor associated neutrophils (TAN) to the granulocytic fraction of myeloid derived suppressor cells (G-MDSC). Each sample was evaluated for changes in the different pathways, and was marked as positive when ≥3 genes were significantly changed to the same direction. In each panel - Right - Pathways/Groups that had more than 5 samples changed. Left - Pathways/Groups that were significantly changed when the groups were compared to each other (p<0.05, corrected). Red – up-regulation; Blue – down-regulation; Black – no change from mean.

**Figure 6:**
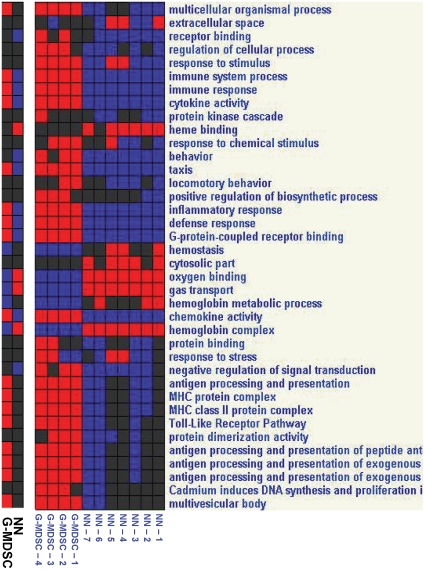
Analysis of pathways and gene groups using Genomica, comparing naïve neutrophils (NN) to the granulocytic fraction of myeloid derived suppressor cells (G-MDSC). Each sample was evaluated for changes in the different pathways, and was marked as positive when ≥3 genes were significantly changed to the same direction. In each panel - Right - Pathways/Groups that had more than 5 samples changed. Left - Pathways/Groups that were significantly changed when the groups were compared to each other (p<0.05, corrected). Red – up-regulation; Blue – down-regulation; Black – no change from mean.

In order to link genes to specific pathways, we evaluated key pathways and gene groups using bioinformatic approaches. Figures 4, 5, and 6 summarize the main differences between the neutrophil populations, comparing TAN and NN (Fig. 4), TAN and G-MDSC (Fig. 5) and G-MDSC and NN (Fig. 6). We further directly evaluated pathways and gene groups related to previously described neutrophil functions and activities. These changes, presented below, are summarized in Table 3.

**Table 3:** Summary of the relative changes in the different neutrophil populations.

*Neutrophil Function*		*NN*	*G-MDSC*	*TAN*
**Structural genes**	Cytoskeleton	+	+	−
	Actin Binding	+	+	−
**Respiratory Burst**	Peroxidase	**++**	**++**	−
	NADPH complex	**++**	+	−/+
	TLR	−	**++**	+
**Granule Proteins**	Primary	**+++**	−	−
	Secondary	**++**	+	−
	Tertiary	**++**	+	−
**Phagocytosis**	Whole group	+/−	+/−	+/−
	Proteolysis/Fibrinolysis	+/−	+/−	+
**Apoptosis**	Whole group	+	+	+
	P53 pathway	−	−	−
	Extrinsic pathway	+/−	+/−	+/−
	Intrinsic (BCL2) Pathway	+	**++**(BH-3)	+/−
	NF-κB – pro apoptotic	+/−	+/−	+/−
	NF-κB – Anti apoptotic	**++**	+/−	+/−
**Immune system**	Whole group	−	+	**++**
	APC genes	−	**++**	**++**
	Cytokine activity	−	+	**++**
	Chemokines	−	+	**+++**

Pathways and gene-groups were evaluated by the Genomica software, and manually from the literature. The data of each neutrophils-function evaluated for each population of neutrophils is presented.

(−) - Most genes in the pathway/group were at background levels.

(+/−) – Some genes of the pathway/group were up-regulated and other down-regulated.

(+) – A related pathway/group was up-regulated (Genomica), or some (>10%) of the genes in the group were up-regulated (manually).

(++) – A related pathway/group was up-regulated (Genomica), and/or a significant portion (>30%) of the genes in the group were up-regulated (manually).

(+++) – A prominent up-regulation of genes in the group/pathway (>50%) was noted.

#### Structural genes

There was down regulation in TAN of pathways related to cytoskeleton organization and biogenesis, and those related to actin binding and polymerization (e.g. Actn-1/4, Raf-1, LCP-1 and Talin-1).

#### Respiratory Burst

There are several gene groups and pathways associated with the respiratory burst [19], [20]. Peroxidase activity genes were down-regulated in TAN compared to NN (Fig. 4). Although the NADPH oxidase complex genes [21] were not significantly changed in the different neutrophil populations, we noted a trend toward down-regulation of several genes in this pathway, i.e. neutrophilic cytosolic factors 1 (p47phox) and 2 (p67phox), as well as RAC-1 and RAC-2 in TAN, compared to NN. Furthermore, we found a significant down-regulation of the fMLP signaling pathway, and its related ERK-1/ERK-2 MAPK signaling pathway, in TAN, compared both to naïve neutrophils (Fig. 4) and to splenic G-MDSC (Fig. 5).

Expression of Toll-like receptor (TLR) signaling genes were low in NN, with marked up-regulation in G-MDSC (Fig. 6). Some of these genes were also up-regulated in TAN compared to NN (i.e. TLR-1, TLR-4 and IRAK-2), but as a group of genes, there were not up-regulated in TAN. There was no change between the different populations in the PMA-stimulated respiratory burst pathway genes (PKC genes).

#### Granule proteins

Neutrophils contain effector granule proteins, stored in primary, secondary and tertiary granules [22]. Although these proteins were not differently expressed as a group, we found that several of these proteins were up-regulated in NN, and to a lesser extent in G-MDSC, compared to TAN where their expression levels were very low. Some of the primary granule genes were significantly and markedly elevated only in NN (i.e. MPO, Proteinase-3 and Cathepsin-G). Interestingly, there was no significant difference in the expression of neutrophil elastase (ELA-2) between the 3 groups of neutrophils. Several secondary granule genes (e.g. MMP-8, CAMP, lactoferrin and NGAL), and most tertiary granule genes (e.g. CD11b, MMP-9 and PGLYRP), were markedly up-regulated in NN, and to some extent also in G-MDSC, compared to TAN.

#### Phagocytosis

We found no clear changes in the pathways and genes related to phagocytosis, as clustered by Theilgaard-Monch et. al. [22]. Interestingly, genes related to proteolysis (e.g. MMP-13/14 and TIMP-1) and fibrinolysis were up-regulated in TAN.

#### Apoptosis

Given data that TAN appear to persist longer than circulating neutrophils [23], we further evaluated some gene-groups related to apoptosis, in both the intrinsic (mitochondrial) and extrinsic (cytosolic, death receptor signaling) pathways [24]. As a whole, there was no significant difference in the apoptosis-related genes and pathways between the 3 populations. All three populations of neutrophils had comparably low levels of genes related to the p53-DNA damage pathway (e.g. p53, MDM-2, ATR, CHEK-1/2 etc.). There was also no clear unidirectional change in the pro-apoptotic and anti-apoptotic regulators of the extrinsic pathway (i.e. TRAIL, Caspase-10). The release of pro-apoptotic proteins from mitochondria, a critical checkpoint in the intrinsic pathway, is regulated by the Bcl-2 family [24]. G-MDSC neutrophils had a clear up-regulation in all pro-apoptotic BH3-only genes, important regulators of this process (i.e. BIM, NOXA, PUMA and BID). We could not find a clear change in this pathway in TAN towards either a pro- or anti-apoptotic direction. In contrast, we found up-regulation of several anti-apoptotic members of the NF-κB family (e.g. IEX-1, SOD-2, GADD-45b and BCL-2A1), in TAN compared to both NN and G-MDSC, whereas the changes in pro-apoptotic members of this family varied.

#### Neutrophils as Antigen Presenting Cells

We found up-regulation in pathways related to the role of neutrophils as antigen presenting cells (APC's) to be a major change in G-MDSC and TAN. When comparing G-MDSC to NN, many of the up-regulated genes and pathways were related to APC function, including general antigen processing and presentation genes, those specifically related to presentation of exogenous peptides (e.g. CD74, Cd1d-1, Psme1, etc.), all six MHC-II protein complex genes (i.e. H2-DMa/DMb1/Aa/Ea/Ab1/Eb1) and Toll-like receptor pathway genes (i.e. TLR-2/6, Fos, Myd88, Jun, etc.) (Fig. 6). The same groups and pathways of genes, except for the Toll-like receptor pathways were also significantly up-regulated when TAN were compared to NN (Fig. 4). There was no significant difference in these pathways between G-MDSC and TAN (Fig. 5). Interestingly, the co-stimulatory molecules CD80 and CD86 were both up-regulated in the G-MDSC compared to NN, and further up-regulated in TAN.

#### Other immune effects and chemokines

Compared to naïve neutrophils, both TAN (Fig. 4) and G-MDSC (Fig. 6) were enriched in pathways related to immune responses and processes as a whole, inflammatory responses, cytokine activity (e.g. TNF-α, IL-1α, IL-1β, IL-12), and receptor binding. Although up-regulation of these immune-related genes were noted in G-MDSC, TAN were further enriched compared to G-MDSC in immune system responses and especially in chemotaxis (Fig. 5).

**Figure 7:**
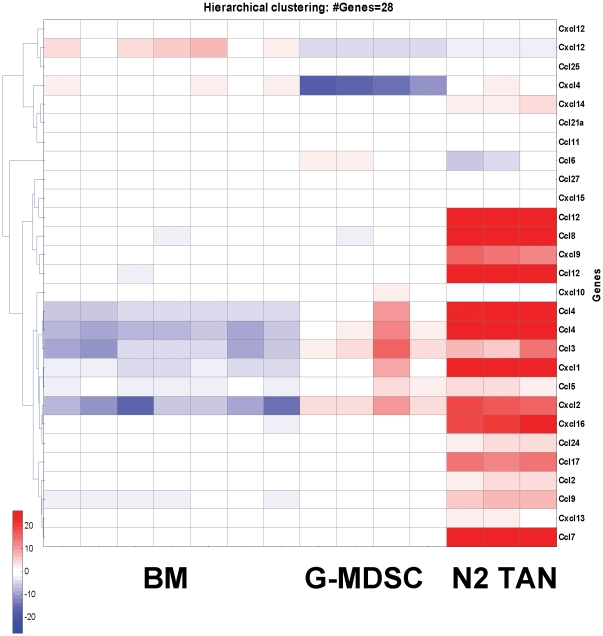
Heatmap comparing the expression of chemokines in the three groups of neutrophils - naïve neutrophils (NN), granulocytic fraction of myeloid derived suppressor cells (G-MDSC) and tumor associated neutrophils (TAN), clearly showing that many chemokines were up-regulated in the TAN group. Red – up-regulation; Blue – down-regulation; White – no change from mean.

One of the major changes in mRNA expression comparing the three neutrophil populations was in chemokine gene expression. Of the 51 genes significantly up-regulated by more than 5-fold in G-MDSC compared to NN (Fig. 2B), 4 were chemokines. Changes were even more prominent when comparing TAN to NN and G-MDSC. For example, of the 86 genes significantly up-regulated by more than 8-fold in TAN compared to G-MDSC, nine were chemokines. Figure 7 is a specific heatmap using all the 25 chemokines present on the microarray. Compared to TAN, NN and G-MDSC were relatively similar to each other in terms of their chemokine profile. CXCL-4 and CXCL-12 were increased in the NN, whereas CCL-3, CCL-4 and CXCL-2 were down-regulated compared with G-MDSC.

In contrast, TAN were quite different than both of these other groups with 13 of these chemokines being markedly up-regulated. These changes included a striking up-regulation of chemoattractants for T-cells (e.g. CCL-17, CXCL-9, CXCL10 and CXCL-16), neutrophils (eg. CXCL-1, CXCL2 and CCL-3), B-cells (CXCL-13) and macrophages (e.g. CCL-2, CXCL-10 and CCL-7).

## Discussion

Although neutrophils are traditionally considered in the context of their anti-bacterial functions, it is becoming increasingly clear that tumor-associated neutrophils (TANs) and their myeloid precursors (peripheral neutrophils and granulocytic MDSC) in the spleen, bone marrow, and blood play an important role in cancer biology [4], [7], [14], [25]. In contrast to the well-described ability of inflammatory neutrophils to engulf bacteria, activate the immune system, and induce tissue damage in infections [26], it appears that myeloid cells can also function as immunosuppressive cells in the context of tumors [27]. This property has been very well described in recent years for the MDSCs found in large quantities in the spleens of tumor-bearing animals [27], [28], [29], [30] and for tumor-associated macrophages which develop an “M2” or tumor-supportive phenotype [1], [3]. Neutrophils make up a significant portion of the inflammatory cell infiltrate in many models of cancer. Previous studies have shown that TAN in untreated tumors can support tumor growth and metastases [31], and this was further supported by our recent work demonstrating that in untreated tumors, TAN develop a pro-tumorigenic (N2) phenotype, largely driven by the presence of TGF-β [4]. The full range of mechanisms responsible for this activity have not yet been elucidated, but neutrophils are known to have pathways that can impact angiogenesis, immune surveillance, as well as secretion of chemokines, cytokines and reactive oxygen species [11], [25].

Since relatively little is yet known about the phenotype of TAN, the goal of the present study was to use an unbiased, discovery-based approach (gene array analysis) to profile and compare the mRNA content of three highly purified granulocytic populations: “naïve” bone-marrow neutrophils from non-tumor-bearing animals (NN) [serving as the “baseline” control population], the granulocytic fraction of myeloid-derived suppressor cells (G-MDSC) in tumor bearing mice, and tumor-associated neutrophils. Our purpose was to gain additional information on the characteristics of TAN, and specifically how much they resembled the other neutrophil populations. A caveat of this study is that we restricted our initial analysis to primarily transcriptomic information. Although we have conducted some functional studies [4], additional experiments, based on this genomic information, will obviously be needed to fully understand the role and functions of TAN.

Using unbiased analyses, our data show that the three populations of neutrophils are significantly different in their mRNA profiles. However, it appears that the NN and G-MDSC are more closely related to each other than to TAN. To better understand the meaning of these differences, we used pathway analyses (using Genomica software, and published gene lists) focusing on several selected pathways and gene groups important for the function and activation of neutrophils including structural genes, respiratory burst, granule proteins, phagocytosis, apoptosis, and immune functions.

NN were relatively enriched in cytoskeleton organization and biogenesis, as well as in pathways related to actin binding and polymerization. This is probably related to the movement needed by neutrophils prior to arriving to their destination [32]. Interestingly these gene pathways are down-regulated in TAN, consistent with their loss of ability to leave the tumor microenvironment after infiltrating the tumor.

The two pathways needed to carry out the “anti-bacterial” functions of neutrophils, granule protein production and the respiratory burst [22], appear to be at their highest levels in the NN. These pathways are progressively lost in the G-MDSC, and even more dramatically in the TAN. TAN show a dramatic down-regulation in mRNA levels of all three groups of granule proteins, which are highly expressed in NN. This finding could be consistent with the work by Shen et. al. showing that TGF-β can inhibit neutrophils degranulation [33]. Given that the two main mechanisms of cell killing by neutrophils (respiratory burst and granule proteins) are both down-regulated in TAN, these findings are compatible with our previously published functional data showing that TAN had low cytotoxic capabilities for tumor cells [4]. An alternative explanation for these findings could be that the mature neutrophils, either G-MDSC or TAN, have finished producing granule contents, and the relevant mRNA are not needed any longer [22], or that the more mature populations have degranulated. If so, these differences may only be a reflection of the fact that NN are less mature. Even if that explains the up-regulation of these mRNAs in NN, there is a further down-regulation in TAN compared to G-MDSC, suggesting a possible effect at the RNA level as well. Again, it is also possible that these differences reflect that G-MDSC are circulatory neutrophils, whereas TAN are in the tissue. The mRNA for neutrophil elastase, a major effector molecule in the activity of neutrophils in general, and specifically in cancer [25], was surprisingly not different between the 3 populations. It is possible that the modifications in the level of this molecule are post-translational, or that it is needed for the proper activity of neutrophils in either compartment. Further research to evaluate actual production and secretion of the different granule proteins is warranted. The only pathway related to respiratory burst where some members were up-regulated in TAN was the toll-like receptor (TLR) family, probably more related to associated immune system changes (see below).

In contrast, we noted no clear changes in the pathways and genes related to phagocytosis, another major function of neutrophils. This was further confirmed by evaluating the genes suggested by Huang et. al. to be phagocytic receptors or genes involved in phagocytic signaling [19] (data not shown).

Despite data suggesting that TAN may be longer lasting cells than circulating neutrophils [23], we found that most genes related to apoptosis were expressed at similar levels. However, we did find that several anti-apoptotic members of the NF-κB family were up-regulated in TAN. NF-κB may be, therefore, an important regulator of the anti-apoptotic machinery in TAN and it is possible that this pathway is responsible for the notable longevity of TAN compare to other neutrophils. Interestingly, we found an up-regulation of all the BH3 pro-apoptotic genes in G-MDSC neutrophils, suggesting that these cells might be especially sensitive to apoptosis-mediated by death receptor ligands.

It has become increasingly clear that the contribution of neutrophils to host defense and natural immunity extends well beyond their traditional role as professional phagocytes [34]. Neutrophils and their myeloid precursors can be induced to express a number of genes whose products lie at the core of inflammatory and immune responses, suggesting a potential role for these cells in orchestrating the sequential recruitment and activation of distinct leukocyte types to the inflamed tissue [35]. Neutrophils from humans and mice are recognized as cellular sources of chemokines in inflammatory responses [35]. “Immunosculpting”, i.e. the crosstalk between immune and tumor cells changing the phenotype of tumor biology, is widely recognized [36]. However, until recently, the role of neutrophils in this cross-talk has been under-estimated. Our study suggests several potential new pathways by which neutrophils can influence both the innate and the adaptive immune system. Our data are also consistent with the suggestion that a potential source for chemokines in tumors are the intratumoral TAN, which constitute a notable percentage of tumor immune cells.

One area of potential importance is in antigen presentation. Accumulating data from the last decade shows that neutrophils can participate in MHC class I and class II restricted antigen presentation, being capable of collecting and cleaving antigens, forming complexes with MHC-II molecules, and expressing co-stimulatory molecules [34], [37], [38]. Our data show that the naïve neutrophils lack many of the gene pathways needed to present antigens, however, both TAN and G-MDSC show increased expression of these genes, suggesting an enhanced capability of functioning as APC's. There was also up-regulation of the co-stimulatory molecules CD80 and CD86. These results are consistent with the abundant data on effects of tumor neutrophils and G-MDSC on T-cells [4], [27], [39], [40]. Interestingly, there was no clear difference in this capability between G-MDSC and TAN, possibly suggesting that this is a fundamental part of activation of neutrophils, regardless of their specific role. An interesting and very important issue, which will require functional studies to elucidate, is whether neutrophil antigen presentation induces T cell activation or anergy. It has been recently shown that mature neutrophils can function as professional antigen-presenting cells capable of priming a Th-1 and Th-17-acquired immune response [38].

The most prominent difference that we found between TAN, and either NN or G-MDSC, was the significant up-regulation of cytokines and chemokines. This change suggests an important role of tumor neutrophils in the recruitment of immunocytes and in the balance between activation and suppression of the immune system. The role of chemokines in the pathogenesis of cancer has been increasingly appreciated [41]. Among the broad group of chemokines whose mRNAs were up-regulated in TAN were the CCL chemokines 2, 3, 4, 8, 12, and 17 and the CXCL chemokines 1, 2, 9, and 16. The up-regulation of chemokines in TAN suggests that upon entering the tumor, TAN have a pivotal role in recruiting other cells of the immune system to the tumor. This is similar to the role that “classical neutrophils” would have in wound healing. At least some of the recruited cells are known to support tumor growth, such as macrophages (by CCL-2 and CCL-7) and T-regulatory cells (by CCL-17) [42]. The increased secretion of CCL-2, CCL-17 and IL-6 was demonstrated at the protein level as well.

Special attention should be given to our data on neutrophil chemoattractants and their chemokine receptors. Ueha et. al have recently summarized the dynamics of myeloid cells, including neutrophils, from the bone marrow to the circulation and into tumors [43]. CXCR-2 and CXCR-4 were shown to cooperatively regulate the release of neutrophils from bone marrow [44]. Whereas the expression of CXCR-2 was not evaluated in this array, CXCR-4 was highly expressed in all 3 neutrophil populations. Its expression, however, was mildly down-regulated in the splenic neutrophils and in TAN. Ueha et. al. further suggest that tumor-infiltration by neutrophils is at-least partly mediated by autocrine CXCL-2 production. Indeed, in our data the expression of CXCL-2 was markedly up-regulated in G-MDSC compared to NN (71-fold), and even more in TAN (188-fold compared to NN). Interestingly, we found similar results in two other known neutrophils chemoattractants – CXCL-1 (increased 14-fold in G-MDSC and 140-fold in TAN compared to NN), and CCL-3 (increased 60-fold in G-MDSC and 76-fold in TAN compared to NN). Unfortunately GCP-2/CXCL-6 was not part of the Illumina array we used, and therefore we could not assess changes in this previously-described [45] neutrophil chemoattractant. It seems therefore, that the neutrophils begin a positive feedback loop by secreting neutrophil chemoattractants that recruit more neutrophils into the tumor, as previously described in infections [46], [47].

It is worth considering the relationship between TAN and the granulocytic fraction of myeloid-derived suppressor cells (G-MDSC) in light of our results. MDSC, a heterogeneous population of immune suppressive cells that are produced at high levels in cancer, are defined in mice on the basis of expression of the surface markers CD11b and GR1 and by their ability to inhibit T lymphocyte activation. The CD11b^+^/GR1^+^ MDSC population is comprised of at least two subsets - granulocytic (Ly6G^+^) and monocytic cells (Ly6C^+^), possibly with different immunosuppressive properties [14]. There is substantial agreement on the immunosuppressive activity of the monocytic MDSC subset. However, there is still contradictory evidence on the role of the granulocytic fraction. Whereas some have shown that granulocytic MDSC have immunosuppression properties similar to the monocytic fraction [28], [29], [30], others have recently demonstrated that they are less immunosuppressive [14], [48], [49]. It has been previously shown that adoptively transferred MDSC can enter tumors and differentiate to mature macrophages (TAM) or neutrophils (TAN) [15], however little is known in animals about whether MDSC leave the spleen and circulate. It is thus not clear whether the majority of TAN are actually G-MDSC that have been attracted to the tumor or whether they are bone marrow/blood-derived neutrophils that were then converted to N2 TAN by the tumor microenvironment, specifically by the high local concentrations of TGF-β [4]. In our previously published work, we saw no effects of TGF-β blockade on the percentage or phenotype of blood neutrophils or splenic MDSC [4]. In the current study, we clearly show that TAN are not “tissue-based G-MDSC”, but are a distinct population of neutrophils, differing markedly in their transcriptomic profile from both NN and G-MDSC. Taken together, these data support the idea that tumor TGF-β (and perhaps other factors) changes only the local “education” of neutrophils. However, the studies described here cannot definitively determine if the cells were recruited from the bone marrow/blood pool of neutrophils or the splenic G-MDSC population.

A possible limitation of our study is that it was performed only in one type of tumor, i.e. the mesothelioma cell line AB12. We did confirm some of the results in a different cell line – the non-small cell lung cancer LKR-M. However further analysis is needed in order to establish the generalization of our data to other tumor systems. It is also possible to argue that since cells were collected at different time points during tumor progression, some of the differences in RNA profiles observed may be due to the time-point at which the cells were isolated (earlier or later during tumor growth) and not their location in the spleen or tumor. Since little is known about the kinetics of the development of TAN, and it is well established that G-MDSC arise only at later time in tumor development [28], we decided to do our comparisons using well-established G-MDSC (associated with larger tumor size) and TAN from established, but not necrotic tumors (less than 500 mm^3^). However, we also confirmed some of our data in key transcripts, using RT-PCR of neutrophils isolated from tumors and spleens at the same time, both at an early and a late time point (Day 14 and Day 21), and showed that similar differences to those seen in the arrays were noted. The important question of the kinetics of the changes in tumor and spleen neutrophils will be addressed in future research.

Our data confirm that the native tumor microenvironment influences the mRNA program of neutrophils. We have previously shown that these cells have pro-tumor characteristics [4]. We have also shown that altering the tumor microenvironment by blocking the effects of TGFβ can further alter the phenotype of TANs to a more “anti-tumor phenotype (N1 TAN). We are currently comparing gene arrays of N1 and N2 TAN and are seeing clear changes in chemokines and cytokines profile between these two populations of neutrophils (manuscript in preparation), suggesting that the specific profile of chemokines secreted is a major characteristic of TAN and a determinant in their polarization. One example is that the T regulatory cell chemoattractant, CCL-17, is up-regulated in N2 versus N1 TAN.

In their recent review on TAN as targets for cancer therapy, Gregory and Houghton argued that changes in TAN are not a switch to a unique transcriptional program, but a heightened state of activation [25]. Borregaard et. al. suggested that circulating neutrophils have two major bursts of transcriptional and protein synthetic activities – one in the bone marrow, producing the granules, and the second upon migration into tissues, resulting mainly in the secretion of cytokines and chemokines [20]. The data we presented here suggests that neutrophils may have a different program if they reside in the spleen, becoming G-MDSC or upon entering the tumor, becoming TAN. If so, these 3 neutrophil populations do have different transcription programs that separate them. It remains to be seen if the differences noted are merely different activation states that can be reversible.

Significant research has recently been done elucidating the important role of myeloid cells in the cancerous process. Our work adds another important layer to the understanding of neutrophils in cancer by further characterizing the different populations of neutrophils induced by tumors and by pointing out major differences between TAN and other neutrophil populations. Further research on the functional role of different pathways and genes up-regulated in TAN and differences between the different subsets of TAN is currently underway.

## Materials and Methods

### Ethics statement

This study was carried out in accordance with the recommendations in the Guide for the Care and Use of Laboratory Animals of the National Institutes of Health. The protocol was approved by the Committee on the Ethics of Animal Experiments of the University of Pennsylvania (Permit Number: 80-2606).

### Animals

Mice were purchased from Taconic Labs (Germantown, NY), and Jackson Labs (Bar Harbor, ME).

### Cell lines

The murine malignant mesothelioma cell line, AB12 was derived from an asbestos-induced tumor in a Balb/C mouse. The murine lung cancer line LKR was derived from an explant of a pulmonary tumor from an activated Kras G12D mutant mouse that had been induced in an F1 hybrid of 129Sv.J and C57BL/6 [50].

### Isolation of the different groups of neutrophils

NN – non-tumor-bearing mice were euthanized, and bone marrow was harvested by flushing the femurs and tibias with HBSS media. Cells were then separated by centrifugation over a 3 layer discontinuous Percoll gradient as previously described [51].

G-MDSC - Mice were injected on the right flank with 1×10^6^ AB12 or LKR tumor cells in the appropriate syngeneic host. The flank tumors were allowed to reach an average size of 500–700 mm^3^ (approximately 21–25 days). Mice were then euthanized, and spleen was harvested. Neutrophils were isolated with microbeads and flow cytometry (see below).

N2 TAN - Mice were injected on the right flank with 1×10^6^ AB12 or LKR tumor cells in the appropriate syngeneic host. The flank tumors were allowed to reach an average size of 250–300 mm^3^ (approximately 12–15 days). Flank tumors were harvested from the mice, minced, and digested with 2 mg/mL DNase I (Sigma, St. Louis, MO) and 4 mg/mL collagenase type IV (Sigma) at 37°C for 1 hour. Neutrophils were isolated with microbeads and flow cytometry (see below). In one set of experiments, both TAN and MDSC were harvested on the same days- Day 14 and 21.

### Isolation of neutrophils from spleens/tumors

Tumors/spleens were harvested, digested, and CD11b^+^ cells were isolated using magnetic beads (Miltenyi Biotec, Germany) per manufacturer's instructions. The CD11b^+^ cells were then “flow sorted” using a Beckman-Coulter EPICS Elite ESP FACS Sorter (Fullerton, CA), isolating CD11b^+^/Ly6G^+^ cells (neutrophils). For that purpose we used Ly6G-PE antibodies (BD Biosciences, Franklin lakes, NJ). In all samples a purity of above 85% neutrophils was achieved.

### Gene microarray

mRNA preparations were extracted from individual mice, and for each “sample”, equivalent amounts of RNA were pooled from 2–4 mice (according to the yield of neutrophils) for the transciptome analysis. We prepared samples from each of the neutrophil populations studied - NN (n = 7), G-MDSC (n = 4) and TAN (n = 4). Samples were processed and hybridized to the Illumina mouse genome bead arrays. Raw data was processed by Bead Studio v.3.0 software. Expression levels were exported for signal and negative control probes. The set of negative control probes was used to calculate average background level for further filtering and background subtraction steps. Average values of the signal probe expression data for the 7 (NN), 4 (G-MDSC) and 4 (TAN) sample arrays were used as a base for normalization and all the arrays were quintile normalized against this base, and filtered to remove non-informative probes. A probe was called non-informative if it had detection p-value>0.05 in all samples or if the maximal ratio between expression values of each two samples was lower than 1.2. The three different groups were compared between each other.

The microarray data complies with the MIAME guidelines, and the data will be deposited in a publicly available database.

### RNA isolation and real-time, reverse transcription-PCR

RNA from tumors/spleens/BM was isolated as above. For each group, a pool of RNA was created by adding the same amount of RNA from each of the samples within the group. Absorbance at 260/280 nanometers for mRNA purity at a ratio above 1.9 was achieved for all samples used. cDNA was made from each pool, RNA levels were normalized to β-actin levels, and quantification of tumor mRNA levels was performed as previously described [4]. Each sample was run in quadruplicate and the experiment was repeated at least once. Primer sequences are given in Table S4.

### Protein validation

In order to validate some of the RNA data at the protein level, neutrophils were isolated as described above. Four million isolated neutrophils were plated in each well of a 12-well plate and covered with 1 ml of medium. After 24 hours, the supernatant was collected, spun, and the level of different proteins was evaluated using ELISA sets for IL-6 (BD Biosciences Pharmingen, San Diego, CA) and CCL-2/MCP-1 (BD Biosciences), and Duoset ELISAs for CXCL-2/MIP-2 and CXCL-10/IP-10 (both from R&D Systems, Minneapolis, MN). For validation of CCL-17, protein was extracted from the neutrophils immediately following isolation, and an immunoblot was performed using anti-CCL-17 Antibody (R&D Systems). TNF-α levels were evaluated by intracellular flow cytometry. BM/Tumor cells were treated with GolgiPlug (BD Biosciences) for 3 h. Thereafter, TNF-α levels were evaluated by intracellular flow cytometry using anti-TNF-α Ab (BioLegend, San Diego, CA) as previously described [52].

### Statistical Analyses

Gene array data was filtered following quintile normalization as described above. Hierarchical clustering was done for evaluation of similarity between samples in the same group, followed by PCA analysis. To compare genes that are different between at least two of the groups, we used one sided ANOVA on the quintile normalization data with appropriate post hoc testing.

Genomica software (http://genomica.weizmann.ac.il/) was used to identify enrichment patterns of experimental signatures associated with the different neutrophils groups [53], [54]. We evaluated about 2300 pathways and gene groups based on the suggested “mouse GO” and “mouse Biocarta” murine gene repositories. Data were log2 transformed and mean centered. Genes whose expression was 2-fold or greater than the mean expression level were scored. Enrichment of over-expressed or under-expressed genes that belong to each tested gene signature was calculated using a hypergeometric test and a false discovery rate (FDR) calculation to account for multiple hypotheses testing (p<0.05, FDR<0.05). The fraction of samples showing significant enrichment for a particular gene signature in each group (BM/N2 TAN/G-MDSC) was calculated.

Heatmaps for lists of genes were composed using two-way hierarchical clustering with normalized Euclidean distance to cluster samples and Spearman correlation distance to cluster genes, with the genes ordered accordingly. For some specific neutrophilic functions, we manually evaluated the specific changes of genes.

For the RT-PCR and FACS studies comparing differences between two groups, we used unpaired Student t-tests. Differences were considered significant when P<0.05. Data are presented as mean+/− SEM.

## Supporting Information

Table S1 **Comparison of the top 15 genes that were changed when comparing naïve neutrophils (NN) to Granulocytic MDSC (G-MDSC).** The genes are shown in order of fold change.
(DOC)

Table S2 **Comparison of the top 15 genes that were changed when comparing naïve neutrophils (NN) to Tumor associated neutrophils (TAN).** The genes are shown in order of fold change.
(DOC)

Table S3 **Comparison of the top 15 genes that were changed when comparing Tumor associated neutrophils (TAN) to Granulocytic MDSC (G-MDSC).** The genes are shown in order of fold change.
(DOC)

Table S4 **The sequences of the primers used for real-time RT-PCR in this manuscript.**
(DOC)
