# Supplementary material for: Transcriptomic Analysis Comparing Tumor-Associated Neutrophils with Granulocytic Myeloid-Derived Suppressor Cells and Normal Neutrophils
Source: PLoS One. 2012 Feb 14;7(2):e31524. doi: 10.1371/journal.pone.0031524 (PMC3279406; doi:10.1371/journal.pone.0031524)
Supplement: Table S1 — Comparison of the top 15 genes that were changed when comparing naïve neutrophils (NN) to Granulocytic MDSC (G-MDSC). The genes are shown in order of fold change. (DOC) [file pone.0031524.s001.doc]

Table S1 – G-MDSC vs. NN – top 15 genes changed

| **AccNumber** | **Gene Symbol** | **P_Value** | **Mean**  **NN** | **Mean**  **G-MDSC** | **Ratio**  **GMDSC - NN** |
| --- | --- | --- | --- | --- | --- |
| NM_008036 | Fosb | 0.012472 | 120 | 8453 | 70.4 |
| NM_009140 | **Cxcl2** | 0.000338 | 222 | 15249 | 68.7 |
| NM_011337 | **Ccl3** | 0.003671 | 267 | 15146 | 56.7 |
| NM_013652 | **Ccl4** | 0.022324 | 231 | 11037 | 47.8 |
| NM_007913 | Egr1 | 5.32E-11 | 1181 | 49282 | 41.7 |
| NM_017466 | Ccrl2 | 1.69E-05 | 457 | 17712 | 38.8 |
| NM_146064 | Soat2 | 3.36E-11 | 203 | 4225 | 20.9 |
| NM_008344 | Igfbp6 | 1.89E-08 | 128 | 2311 | 18.1 |
| NM_009397 | Tnfaip3 | 0.003276 | 508 | 8982 | 17.7 |
| NM_007443 | Ambp | 3.32E-12 | 124 | 1989 | 16.1 |
| NM_030701 | Gpr109a | 3.50E-07 | 1045 | 16452 | 15.7 |
| NM_001081957 | LOC100034251 | 2.25E-08 | 1790 | 26456 | 14.8 |
| NM_030612 | Nfkbiz | 1.99E-06 | 2057 | 29746 | 14.5 |
| NM_020001 | Clec4n | 1.04E-09 | 285 | 4105 | 14.4 |
