# Supplementary material for: Transcriptomic Analysis Comparing Tumor-Associated Neutrophils with Granulocytic Myeloid-Derived Suppressor Cells and Normal Neutrophils
Source: PLoS One. 2012 Feb 14;7(2):e31524. doi: 10.1371/journal.pone.0031524 (PMC3279406; doi:10.1371/journal.pone.0031524)
Supplement: Table S2 — Comparison of the top 15 genes that were changed when comparing naïve neutrophils (NN) to Tumor associated neutrophils (TAN). The genes are shown in order of fold change. (DOC) [file pone.0031524.s002.doc]

Table S2 – TAN vs. NN – top 15 genes changed

| **AccNumber** | **Gene Symbol** | **P_Value** | **Mean**  **NN** | **Mean**  **TAN** | **Ratio**  **TAN - NN** |
| --- | --- | --- | --- | --- | --- |
| NM_013652 | **Ccl4** | 1.29E-13 | 194 | 58938 | 303.3 |
| NM_023516 | 2310016C08Rik | 4.91E-07 | 153 | 30720 | 200.23 |
| NM_009140 | **Cxcl2** | 2.31E-10 | 202 | 38054 | 188.73 |
| NM_011331 | **Ccl12** | 1.74E-05 | 93 | 15012 | 161.43 |
| NM_008176 | **Cxcl1** | 1.77E-07 | 94 | 12379 | 131.53 |
| NM_021443 | **Ccl8** | 1.09E-09 | 103 | 12448 | 120.93 |
| NM_007669 | Cdkn1a | 6.87E-07 | 154 | 15768 | 102.2 |
| NM_013654 | **Ccl7** | 1.23E-05 | 83 | 7957 | 95.5 |
| NM_011337 | **Ccl3** | 7.41E-10 | 232 | 17963 | 77.6 |
| NM_007482 | Arg1 | 5.63E-05 | 81 | 5827 | 72 |
| NM_023118 | Dab2 | 4.07E-06 | 147 | 9721 | 66.3 |
| NM_008605 | Mmp12 | 5.02E-06 | 86 | 5268 | 61.4 |
| NM_021893 | Pdcd1lg1 | 1.94E-07 | 256 | 15544 | 60.7 |
| NM_007498 | Atf3 | 0.000271 | 115 | 6738 | 58.3 |
