# Supplementary material for: Transcriptomic Analysis Comparing Tumor-Associated Neutrophils with Granulocytic Myeloid-Derived Suppressor Cells and Normal Neutrophils
Source: PLoS One. 2012 Feb 14;7(2):e31524. doi: 10.1371/journal.pone.0031524 (PMC3279406; doi:10.1371/journal.pone.0031524)
Supplement: Table S3 — Comparison of the top 15 genes that were changed when comparing Tumor associated neutrophils (TAN) to Granulocytic MDSC (G-MDSC). The genes are shown in order of fold change. (DOC) [file pone.0031524.s003.doc]

Table S3 – TAN vs. G-MDSC – top 15 genes changed

| **AccNumber** | **Gene Symbol** | **P_Value** | **Mean**  **G-MDSC** | **Mean**  **TAN** | **Ratio**  **TAN - NN** |
| --- | --- | --- | --- | --- | --- |
| NM_027521 | 6330406L22Rik | 0.000349 | 59 | 13267 | 223.7 |
| NM_021443 | **Ccl8** | 0.001583 | 107 | 17476 | 163.0 |
| NM_011331 | **Ccl12** | 1.03E-06 | 117 | 16896 | 144.0 |
| NM_007482 | Arg1 | 0.013307 | 92 | 9722 | 106.0 |
| NM_013654 | **Ccl7** | 5.28E-05 | 109 | 10550 | 96.9 |
| NM_023118 | Dab2 | 3.45E-05 | 133 | 11360 | 85.3 |
| NM_009890 | Ch25h | 2.13E-06 | 94 | 6387 | 67.7 |
| NM_008607 | Mmp13 | 0.000153 | 160 | 9393 | 58.5 |
| NM_009263 | Spp1 | 1.42E-08 | 379 | 15790 | 41.7 |
| NM_019932 | **Cxcl4** | 1.53E-05 | 164 | 6780 | 41.3 |
| NM_053113 | Ear11 | 5.55E-05 | 142 | 4701 | 33.1 |
| NM_023516 | 2310016C08Rik | 5.20E-05 | 982 | 31510 | 32.1 |
| NM_023158 | **Cxcl16** | 2.56E-06 | 200 | 6368 | 31.8 |
| NM_031254 | Trem2 | 3.06E-07 | 171 | 5249 | 30.8 |
