# Supplementary material for: Transcriptomic Analysis Comparing Tumor-Associated Neutrophils with Granulocytic Myeloid-Derived Suppressor Cells and Normal Neutrophils
Source: PLoS One. 2012 Feb 14;7(2):e31524. doi: 10.1371/journal.pone.0031524 (PMC3279406; doi:10.1371/journal.pone.0031524)
Supplement: Table S4 — The sequences of the primers used for real-time RT-PCR in this manuscript. (DOC) [file pone.0031524.s004.doc]

**Table S4 – Primer sequences.**

| **Name of primer** | **Sequence (5' to 3')** |
| --- | --- |
| **TNFα-5'** | ACCACGCTCTTCTGTCTACT |
| **TNFα-3'** | AGGAGGTTGACTTTCTCCTG |
| **CXCL1-5’** | CCGAAGTCATAGCCACACTCAA |
| **CXCL1-3’** | GCAGTCTGTCTTCTTTCTCCGTTAC |
| **CXCL2-5’** | AGACAGAAGTCATAGCCACTCTCAAG |
| **CXCL2-3’** | CCTCCTTTCCAGGTCAGTTAGC |
| **CCL3-5'** | ACCATGACACTCTGCAACCA |
| **CCL3-3'** | TCAGGCATTCAGTTCCAGGT |
| **CCL17-5'** | TCACTTCAGATGCTGCTCCT |
| **CCL17-3'** | TCACCAATCTGATGGCCTTC |
| **CXCL-10-5’** | CAATGATCTCAACACGTGGG |
| **CXCL-10-3’** | GTCTGAGTGGGACTAAGGG |
| **CCL2-5'** | CAGGTCCCTGTCATGCTTCT |
| **CCL2-3'** | GTCAGCACAGACCTCTCTCT |
| **CCL5-5'** | ACCATGAAGATCTCTGCAGC |
| **CCL5-3'** | TGAACCCACTTCTTCTCTGG |
| **ICAM-1-5'** | GAAGCTTCTTTTGCTCTGCC |
| **ICAM-1-3'** | AGCAGTACTGGCACCAGAAT |
| **VEGF-5’** | ATGGATGTCTACCAGCGAAG |
| **VEGF-3’** | CTGAACAAGGCTCACAGTGA |
| **MMP9-5’** | CTGCATTTCTTCAAGGACGG |
| **MMP9-5’** | AAGTCGAATCTCCAGACACG |
